# A New Process of Mummification

**Published:** 1868-09

**Authors:** 


A New Process of Mummification.—Some few months since the French and Italian papers were full of descriptions of Prof. Marini's discoveries of the process of mummification, petrifaction, and his methods for restoring mummified bodies, or portions of the same, to their original volume, color and appearance. The English papers pooh-poohed the accounts; decided *a priori* that they must be exaggerated. Our curiosity was aroused, and, availing ourselves of an introduction, we visited the professor in his modest abode, and spent an afternoon in examining his specimens and in listening to his explanations; and as his labors bid fair not only to advance science, to protect students of anatomy from its attendant dangers, but also to alleviate human sufferings, we felt no doubt that the subject is one fraught with interest for all classes and all countries.

The first specimen the professor showed us was the foot of an Egyptian mummy, of which the half remained in its mummified condition; the other half was restored to its normal color, form, dimensions, and suppleness. Shutting the window and holding a candle behind the foot, you see the transparency of the soft, and shadows of the hard substances. Another foot, perforated by a ribbon and sealed, with the inscription: "Pied à l'état sec vu le 29 janvier, 1868, Nelaton," was presented supple, fresh, and of normal color, and on the reverse of the parchment was written: "Ce même pied examinè le 26 fèvrier, a repris sa souplesse assez complètement pour que j'aie pu dissèquer assez facilement le muscle abducteur du cinquième orteil, Nelaton." Next came a hand, on which was written: "[illegible]aris, 14 novembre, 1864. La main est à l'ètat sec, C. Sapey," and lower down: "Le 25 novembre, 1864. Cette main a repris sa flexibilité et tous les caractères qu'elle prèsente à l'ètat frais, C. Sapey."

On this hand Nelaton experimented. Hitherto it had been impossible to inject the arteries with wax or other substances used in anatomical studios, on account of the salts which form in the arteries themselves, preserved by systems hitherto known; whereas, by Marini's system a corpse may be injected with the substances necessary to facilitate a perfect study of all the arteries, even after many years of preservation.

Considering the number of students who annually lose life or limb in the act of dissecting, if Marini's system shall denude the corpse of its venom and present a harmless subject to the student, for this gain alone humanity will have cause for gratitude. According to some of the most celebrated anatomists this point is already gained. "Where," writes Professor Corrado Tommasi, "Marini has reached the summit of art in anatomical preparations, is in the process by which he obtains the preservation of animal tissues in a fresh state. Among his preparations he possesses an arm detached in September, 1864, from a corpse in the *Ecole pratique* of Paris (bearing the seal and signature of Professor Sapey) which seems freshly severed from the body to which it belonged. All the organs which enter into the composition of this member—skin, tissue, nerve, muscle, etc.—have preserved the size, color, consistency, and suppleness of a fresh corpse not yet rigid. This arm may be desiccated to-day, and be used for an anatomical demonstration precisely as it was used three years since in the Practical School of Paris.

"Nor is this process of Marini applicable solely to partial fragments of the human form, since he preserved for an entire year in a fresh state the corpse of Professor Martini, of Cagliari, previous to petrifying it, and four months after death dressed it, and had a photograph taken so perfect and expressive as to delight all those to whom Martini was known. In the experiments performed before the commission of the Medical Academy of Florence, Marini arrested the already commenced putrefaction of the corpse of a four-years child, restoring to it all the appearance of freshness, which it still maintains despite the intense heat of last year's summer. The brain, the spinal marrow, the most delicate tissues of pathological formation, are perfectly preserved with all their normal appearances, and apparently for ever. A microscopic examination of all these preparations demonstrates that the elements of the tissues have suffered no variation of form or dimensions; possibly one observes a slight opacity, especially where albuminous matters abound. But there is no coagulation even of these substances, no granulous appearance in the contents of the cellular elements or of their derivations."

The photograph of Professor Martini to which Tommasi alludes I enclose, leaving it to those who see it to say whether it appears to have been taken *four months after* death, as the acts and documents of the municipality of Cagliari prove beyond all doubt that it was.

These two processes of *transitory mummification*, and the preservation of animal tissues in a fresh state, are obtained by the immersion of the body in a liquid composed of *vegetable* substances whose component parts are Marini's secret, jealously kept hitherto, to the displeasure of the medical faculty, who fear that, like Segato, he will take his discovery with him into the grave. On this point I ventured to question him, and he assures me that he has no such intention, that he has already declared to the commission of the Medical Academy of Florence that he will reveal his secret to them, and afterwards to the public, as soon as they shall present their report on the experiments of which they have been witnesses. As usual, Marini has not proved a prophet in his own country. Even as in the case of the brothers Lollini, it was left for the Professors Tardieu and Nelaton to proclaim to the world the excellence of their surgical instruments, and to award them the first-class gold medal at the Paris Industrial Exposition, while Italian surgeons persisted in exalting the superiority of the Charrière manufactory, and the Italian Government in supplying the army and navy from the same, so it was to Nelaton and his *confrères* that Marini had to look for any official recognition of his wonderful discovery. But a worthier motive than this natural pique keeps him silent yet. His experience in the substances best adapted to preserve inanimate matter from decay led Marini to experiment on animate matter affected, if we may so express it, by premature decay, by death in life.

In the hospital of S. Maria Nuova, in Florence, with the consent of the attendant surgeons, Marini applied his lotion to several patients affected with cancer, ulcer and similar diseases. In the first place, this lotion was applied to a foot covered with ulcerated and bleeding sores, or, as the *Sperimentale* expresses it, large *fungous masses abundantly sanguineous.* The ordinary method adopted by Dr. Rosati and by his predecessor, Professor Paoli, had not succeeded in bringing about any useful modification of the wound which could point to the hope of cicatrization of the surface. On the contrary, the rapid reappearance of the fungi after their removal pointed to the necessity of operation. Marini's liquid was tried for a month. Rapid and profound were the modifications that ensued. At the end of the month two-thirds of the affected part were cicatrized. Professor Marini absenting himself from Florence, the application was discontinued. In a few days the wound broke out afresh, nor did the cicatrization proceed one step. The lotion again applied, the patient has recovered all that he lost during the professor's absence.

In the case of another patient the liquid produced, in a very short space of time, cicatrization of a varicose wound which had defied all other treatment. After the quotation of other cases by Dr. Rosati, one of which he requested the members of the society from whose *procés-verbal* we quote to watch and examine, Dr. Billi certified that he had applied the Marini liquid to one of his own relations affected with cancer of the tongue; that said cancer, which had proceeded with frightful rapidity for two months, and which, according to the first surgeons, could not be extirpated, had been *arrested* by the application of Marini's liquid. Other cases are still under cure. Two women affected with internal cancer are now being treated by him in the hospital, and but last week he was summoned to attend the sister of the Syndic of Naples, on whom his liquid has already produced the desired effect. The surgeons, even those who apply his lotion, are not on good terms with him, because he will not reveal the ingredients of which it is composed. He justifies himself by saying, that when he first applied the lotion to cancer, especially to internal cancer, it had the great defect of fostering, rather than stopping hemorrhage; that for this defect he has found the remedy, and that when he has brought his liquid to what he considers perfection, he will, in his own way and on his own terms, reveal his secret.

It is to the curative results of Marini's discovery, to the cure of cancer and of hospital gangrene, now in course of experiment, that we attach more importance than to any other, singular and interesting as all are. His preservation of animal food is also a useful invention; meat desiccated in one year has been eaten the next, as his liquid has no smell and no deleterious effects.

His petrifactions are the most curious, though perhaps of least practical utility, of all his discoveries, as any animal substance once reduced to a state of petrifaction cannot be restored to its fresh state as when only mummified or dried. He showed us petrified livers of human beings and animals, a petrified medal of Garibaldi's blood, a petrified rabbit, etc., etc., strike them with a hammer, and they give the ring of stone, and, like stone, they break into fragments if hit hard enough; but they are not as cold as stone, and if you hold a light behind them, they are transparent at the edges. As his last specimen, the professor uncovered a small table standing in the middle of the room, which to all appearances, was made of Florentine mosaic encrusted in the ordinary cement. Pointing to the bright red bits, he said: "That is human blood, that bullock's, that fowl's. Those purple bits are liver; those, lights; those, lungs; that is bile; the cement of the whole is human brain." We laid our hands on this extraordinary conglomeration and found it less cold than marble, but to the touch of the hammer it gave forth a similar sound.

A similar, though far more elaborate, table was presented to the Emperor of the French, who was deeply interested in Marini's discoveries, which he caused to be thoroughly investigated by Professor Nelaton.

In this table, composed of blood, bile, liver, tissues, brain, four human ears are encrusted; in the centre is poised a woman's foot, which preserves its natural color and transparency, the whole bearing an even and brilliant polish. It has been sent by the Emperor to the Orfila Museum, whence it is to be transferred to the Museum of Natural History.—*Florence (Italy) correspondence of "The Nation."*

